# Alternative and Natural Therapies for Acute Lung Injury and Acute Respiratory Distress Syndrome

**DOI:** 10.1155/2018/2476824

**Published:** 2018-05-16

**Authors:** Vipul J. Patel, Sreeja Biswas Roy, Hiren J. Mehta, Myungsoo Joo, Ruxana T. Sadikot

**Affiliations:** ^1^Division of Pulmonology, Norton Thoracic Institute, St. Joseph's Hospital and Medical Center, Phoenix, AZ, USA; ^2^Division of Pulmonary and Critical Care Medicine, Department of Medicine, University of Florida, Gainesville, FL, USA; ^3^Division of Applied Medicine, School of Korean Medicine, Pusan National University, Yangsan 626-870, Republic of Korea; ^4^Department of Veterans Affairs, Atlanta VAMC, Emory University School of Medicine, Atlanta, GA, USA; ^5^Division of Pulmonary and Critical Care Medicine, Department of Medicine, Emory University School of Medicine, Atlanta, GA, USA

## Abstract

**Introduction:**

Acute respiratory distress syndrome (ARDS) is a complex clinical syndrome characterized by acute inflammation, microvascular damage, and increased pulmonary vascular and epithelial permeability, frequently resulting in acute respiratory failure and death. Current best practice for ARDS involves “lung-protective ventilation,” which entails low tidal volumes and limiting the plateau pressures in mechanically ventilated patients. Although considerable progress has been made in understanding the pathogenesis of ARDS, little progress has been made in the development of specific therapies to combat injury and inflammation.

**Areas Covered:**

In recent years, several natural products have been studied in experimental models and have been shown to inhibit multiple inflammatory pathways associated with acute lung injury and ARDS at a molecular level. Because of the pleiotropic effects of these agents, many of them also activate antioxidant pathways through nuclear factor erythroid-related factor 2, thereby targeting multiple pathways. Several of these agents are prescribed for treatment of inflammatory conditions in the Asian subcontinent and have shown to be relatively safe.

**Expert Commentary:**

Here we review natural remedies shown to attenuate lung injury and inflammation in experimental models. Translational human studies in patients with ARDS may facilitate treatment of this devastating disease.

## 1. Introduction

Acute lung injury (ALI) and acute respiratory distress syndrome (ARDS) are associated with high morbidity and mortality rates [1–3]. These disorders are characterized by rapid-onset respiratory failure, severe hypoxemia, and decreased static respiratory system compliance [2]. A recent consensus-based definition (i.e., the Berlin definition) has proposed the subdivision of ARDS into three categories based on degree of hypoxemia and has urged practitioners to drop the term “acute lung injury” [4, 5]. Increased severity of ARDS is associated with poorer prognosis and higher mortality [6]. ARDS results from uncontrolled acute inflammation and dysfunction of endothelial and epithelial barriers of the lung, and an excessive transepithelial leukocyte migration, leading to the loss of alveolar-capillary membrane integrity and overproduction of proinflammatory cytokines. The pathogenesis of ARDS involves activation of both immune and structural cell types. Immune cells implicated in ARDS include macrophages and neutrophils [7–9], as well as lymphocytes and platelets [7, 9]. The inflammatory response in ALI and ARDS is initiated, amplified, and modulated by a complex network of cytokines and other proinflammatory molecules produced by a variety of cell types in the lungs, including fibroblasts, epithelial cells, and inflammatory cells [10].

Endothelial injury is an underlying cause of increased permeability and pulmonary edema in ALI and ARDS, but epithelial injury also plays an important role in their development. Endothelial activation may also lead to obstruction or destruction of the pulmonary vasculature [11]. Injury to alveolar type II cells contributes to surfactant abnormalities [8]. The hallmark of therapy for ALI and ARDS is supportive care [12]. Despite an increased understanding of its molecular pathogenesis, specific therapies have yet to be developed for ARDS [13, 14].

Contemporary approaches to develop drug therapies have not been productive. In particular, blockade of single cytokines and chemokines have failed to improve outcomes because of the complex pathogenesis and nature of ARDS. Therefore, defining the contribution of proximal signaling pathways that amplify the inflammatory response and developing therapies to specifically block them is an attractive approach, one that may limit injury and inflammation associated with this devastating disease. Intracellular signaling pathways triggered by diverse pattern-recognition receptors converge on signaling hubs, including transcription factors nuclear factor *κ*B (NF-*κ*B), interferon regulatory factor families, STAT, and AP-1. There is also simultaneous activation of oxidant and antioxidant pathways, particularly in innate immune cells.

Nuclear factor erythroid 2-related factor 2 (Nrf2), a member of the cap'n'collar family of basic leucine zipper transcription factors, provides a key antioxidant response. Most widely studied experimental models use lipopolysaccharide (LPS), an endotoxin of gram-negative bacteria. More recently, the roles of other molecules (e.g., danger-associated molecular pattern molecules [DAMPS]), intracytoplasmic receptors (e.g., nod-like receptors [NLRs]), amplifiers (e.g., triggering receptors expressed on myeloid cells-1 [TREM-1]), and several others are being recognized. The detailed molecular mechanisms of lung injury and ARDS have been reviewed extensively in several recent publications [7, 9, 15].

Since its initial description in 1967, little progress has been made in the development of novel therapies for ARDS. To date, no pharmacological agents have demonstrated efficacy in preventing ARDS or improving its symptoms, and the morbidity and mortality continue to be significant [6, 16, 17]. Hence, ARDS represents an unmet medical need, and the need to develop new therapies to treat patients with this condition is urgent. Certain natural remedies have been shown to inhibit multiple inflammatory pathways associated with ALI/ARDS at a molecular level and therefore may be effective in ARDS treatment. Here we review some of the natural products that have been studied in lung inflammation. After summarizing some of the key inflammatory pathways that play a role in lung injury, we will discuss natural products that target these pathways.

## 2. Inflammatory Pathways That Contribute to Pathogenesis of Acute Lung Injury

Inflammation is an important component of ALI and ARDS, as inflammation is what damages the respiratory membrane. Most inflammatory cells, including macrophages and neutrophils, release inflammatory cytokines in response to various stimuli. LPS, a main component of the outer membranes of gram-negative bacteria, has been identified as a key risk factor for ALI and ARDS. LPS binds to Toll-like receptor 4 (TLR4), which induces activation of intracellular pathways. Ligand binding to TLR4 induces the recruitment and activation of adaptor proteins through the Toll/interleukin- (IL-) 1 receptor (TIR) domain. Recruitment of the adaptor protein myeloid differentiation primary response gene 88 (MYD88) to the receptor complex will only occur if the TIR domain contains adaptor protein (then called TIRAP, or MAL). MYD88 recruits IL-1 receptor-associated kinase 4 (IRAK4), which forms an active complex capable of recruiting the tumor necrosis factor (TNF) receptor-associated factor 6 (TRAF6).

**Figure 1:**
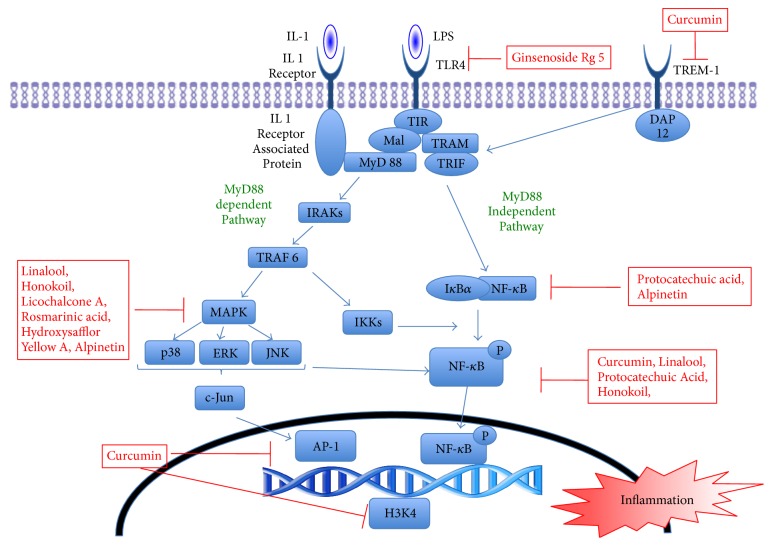
Intracellular signaling pathways associated with inhibition of the nuclear transcription factor-kappa B (NF-*κ*B).

Activation of TRAF6 leads to activation of I*κ*B kinase (IKK) enzyme complex and regulatory scaffold proteins [18]. The associated pathway of mitogen-activated protein kinase (MAPK) also leads to activation of the nuclear transcription factor NF-*κ*B. Three types of MAPKs have been studied: (1) extracellular signal-regulated protein kinase (ERK), (2) c-jun n-terminal kinase (JNK), and (3) p38 MAPK. Inhibition of any of these MAPK pathways significantly decreases TNF-*α* production by LPS (Figure 1).

NF-*κ*B plays a central role in intracellular inflammatory pathways. The most predominantly characterized NF-*κ*B complex is the p50/p65 heterodimer. In most cells, NF-*κ*B remains inactive in the cytoplasm in a complex with any of the family of inhibitory I*κ*B proteins [7]. Activation of inflammatory pathways induces phosphorylation, ubiquitination, and proteasome-mediated degradation of the I*κ*B protein, followed by translocation of NF-*κ*B to the nucleus and regulation of gene expression through binding to the* cis*-acting NF-*κ*B element. The tyrosine phosphorylation of p65 NF-*κ*B efficiently modulates transcription activity. Activation of NF-*κ*B leads to expression of transcription of adhesion molecules, chemokines, colony-stimulating factors, and other cytokines necessary for inflammatory responses.

TREM-1 belongs to the TREM superfamily of receptors expressed on monocytes and neutrophils. Although the precise ligands for TREM-1 have not been identified, it is significantly upregulated by various TLR ligands, including lipoteichoic acid (ligand for TLR2), polyinosinic-polycytidylic acid (ligand for TLR3), and LPS (ligand for TLR4).

These receptors activate downstream signaling pathways with the help of an adaptor molecule, TYRO protein tyrosine kinase binding protein (DAP12). The activation of TREM-1 synergizes with the effects of the TLR ligands and amplifies the synthesis of inflammatory cytokines. This interaction then leads to the activation of NF-*κ*B and to the release of proinflammatory TNF-*α*, IL-12, IL-1, IL-6, IL-8, and anti-inflammatory cytokines IL10 and TGF-*β*. Recent studies also suggest that activation of TREM-1 is modulated by prostaglandins [47] and that it prolongs survival of activated macrophages [48]. We recently used a novel nanomiceller approach to show that blocking TREM-1 attenuates LPS-induced lung injury in a murine model. Additionally, we have shown that curcumin inhibits the binding of p65 to TREM-1 promoter in response to LPS—which enhances the anti-inflammatory effects of curcumin [19].

## 3. Natural Products That Target the Inflammatory Pathway

Clinically used anti-inflammatory drugs have several disadvantages, including adverse effects and a high cost of treatment. Since ancient times, traditional medicines and phytopharmaceuticals have been used to treat inflammation and other disorders, especially in the Asian subcontinent. Such treatments are natural products, and this affords us the valuable opportunity to identify their bioactive compounds, which could ultimately translate into development of new drugs for treatment of inflammatory diseases. The potential of these compounds to attenuate inflammation in the lungs has been studied in cell cultures and animal models. Several studies have focused on investigating natural compounds that can inhibit TLR signaling pathways, particularly through inhibition of NF-kB.

Tables 1 and 2 summarize some of the natural products that have been shown to attenuate inflammation and have been studied* in vitro *and* in vivo *in experimental models of lung injury. Kim et al. [21] showed that Ginsenoside Rg5, a rhizome extract, significantly decreased inflammation in ALI and ARDS models by interacting with TLR4 receptor. Alpinetin, derived from seeds of* Alpinia katsumadai Hayata*, inhibits phosphorylation of the I*κ*B*α* protein, eventually decreasing activation of NF-*κ*B [22]. Additional studies have shown that alpinetin specifically inhibits phosphorylation of p38 and ERK-associated pathways. An* in vitro *study showed that protocatechuic acid (PCA), a benzoic acid derivative, inhibits degradation and phosphorylation of I*κ*B*α*, thereby decreasing NF-*κ*B activation [23]. Several naturally occurring products have been shown to attenuate inflammation by inhibiting phosphorylation of p38 and ERK pathways. Chu et al. [24] reported that Licochalcone A (LicoA), found in the root of Xinjiang licorice, suppressed NF-*κ*B and p38/ERK MAPK signaling in a dose-dependent manner.

LicoA has also been shown to inhibit vascular smooth muscle proliferation by suppressing platelet-derived growth factor-induced activation of the ERK1/ERK2 pathway. Rosmarinic acid, a natural prolyl oligopeptidase inhibitor, increases superoxide dismutase (SOD) activity and suppresses ERK/MAPK signaling [25].

Additionally, rosmarinic acid has other effects, such as inhibition of the complement cascade, which may also contribute to its protective effects. Hydroxysafflor yellow A (HSYA) inhibits MAPK, thereby inhibiting NF-*κ*B activation [26, 27]. Linalool, a major volatile component of essential oils in several aromatic plant species, demonstrated anti-inflammatory capability in* in vitro *and* in vivo *models of ALI/ARDS [28]. Patchouli alcohol has also been shown to have anti-inflammatory effects on mouse ALI models by inhibiting IkB-alpha and p65 NF-*κ*B phosphorylation induced by LPS [29]. Bai et al. [30] showed that shikonin, a natural pigment, suppressed LPS-induced COX 2 and iNOS activation by downregulating NF-*κ*B activation.

Table 2 lists some of the natural products that inhibit inflammation in ALI or ARDS models. Honokiol, a component of a Chinese tree, decreases production of early-phase cytokines (e.g., HMGB1) in mice models. It also inhibits protein kinase C-*α* and MAPK [31]. Isoforskolin (ISOF) has been shown to prevent LPS-induced ALI development in pretreated animal models [32]. ISOF is an effective adenylyl cyclase activator that causes increased intracellular cyclic adenosine monophosphate (cAMP), which has attenuated in* in vitro *LPS-induced ALI. Caffeic acid phenethyl ester (CAPE), an extract of propolis, has exhibited antioxidant qualities [34], as well as anti-inflammatory effects by modulating the arachidonic acid (AA) cascade. It also inhibits Na+/K^+^ ATPase activity in LPS-induced ALI models. Ruscogenin has been shown to inhibit tissue factor expression and iNOS and NF-*κ*B activation [35]. In rats, the bark extract of* Bathysa cuspidata *attenuates ALI-induced by paraquat by reducing lipid and protein oxidation and preventing a reduction in catalase and SOD activity [36]. Shin et al. [37] showed that a traditional herbal remedy,* Callicarpa japonica Thunb *(CJT), inhibited LPS-induced inflammation by reducing iNOS expression and interleukin-6* in vitro *and* in vivo.*

The Chinese herbal formula Huang-Lian-Jie-Du-Tang (HLJDT) comprises* Rhizoma coptidis, Radix scutellariae, Cortex phellodendri, *and* Fructus gardeniae*. In rats with LPS-induced ALI, HLJDT dose-dependently reduced the number of leukocytes adhering to the endothelium and decreased the expression of VCAM1 in lung venules.* In vitro*, HLJDT inhibited NF-*κ*B nuclear translocation in endothelial cells [49].

As noted above, TREM-1 is a prolific amplifier of TLR-induced inflammatory responses. Curcumin (or diferuloylmethane), a natural product found in tumeric, has been shown to decrease inflammation by inhibiting multiple proinflammatory pathways and activating anti-inflammatory pathways [20]. We have shown that curcumin inhibits the expression of TREM-1* in vitro *in primary bone marrow derived macrophages and* in vivo *in the lungs of mice with sepsis. Chromatin immunoprecipitation assay confirmed that curcumin inhibits the binding of p65 to TREM-1 promoter in response to LPS. Furthermore, we showed that curcumin attenuated methylation and acetylation of histone 3 and histone 4 (H3K4) by inhibiting p300-HAT, a key epigenetic element known to activate transcription of the genes that regulate inflammation [19].

Together these studies highlight the potential of several natural compounds that can attenuate lung inflammation by pleiotropic actions and that inhibit key signaling components and amplifiers of TLR pathways. Although some of these studies provide proof-of-principle data in cell and preclinical models, translation of these studies to human clinical trials is lacking.

## 4. Nuclear Factor Erythroid 2-Like 2 (Nrf2): An Anti-Inflammatory Transcription Factor

**Figure 2:**
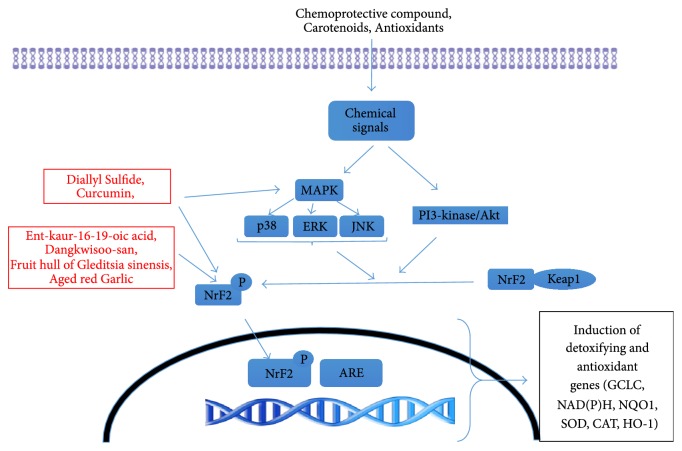
Cellular pathways involved in regulating the phosphorylation of nuclear factor erythroid 2-related factor 2 (Nrf2) and antioxidant response element- (ARE-) mediated antioxidant gene expression.

Oxidative stress also plays a key role in the development of ALI and ARDS. Tissue homeostasis requires that an intricate, delicate balance between oxidants and antioxidants be maintained. Any disruption in this checks-and-balances system can lead to harmful consequences, particularly in the setting of lung injury. Some cells, including pulmonary macrophages, express various proteins that scavenge reactive oxygen species. One of the key transcription factors that induces these proteins is Nrf2, a member of the cap'n'collar family of basic leucine zipper transcription factors. The inactive form of Nrf2 remains bound in cytosol by Kelch-like ECH-associated protein 1 (KEAP1). In pulmonary macrophage activation, Nrf2 binds to its* cis*-acting antioxidant response element (ARE) sequence, resulting in expression of various phase 2 detoxification genes, including glutamate-cysteine ligase, catalytic subunit (GCLC), NAD(P)H, quinone-1 (NQO1), SOD, catalase (CAT), glutathione peroxidase (GPx), and heme oxygenase-1 (HO-1). Studies have demonstrated that phosphatidylinositol 3-kinase/Akt and various MAPKs (e.g., ERK, JNK, and p38) are involved in regulating the phosphorylation of Nrf2 and ARE-mediated antioxidant gene expression (Figure 2) [50].

Table 3 lists natural products that have been shown to decrease inflammation through this pathway. Dangkwisoo-san (DS), a Korean herbal remedy, has been shown to activate Nrf2 and induce Nrf2-regulated genes (including GCLC, NQO1, and HO1) in* in vitro *studies [40]. DS is thought to activate Nrf2 by dissociating KEAP1 from Nrf2.

Three separate herbal product derivatives, ent-kaur-16-19-oic acid (KA) [41], the fruit hull of* Gleditsia sinensis *(FGS) [42], and Carthami Flos (CF) [43], have been shown to activate Nrf2 and to induce Nrf2-regulated gene expression in* in vitro *macrophage cell lines. CF in particular attenuated neutrophilic lung inflammation in mice, in the presence of Nrf2 [43]. Diallyl sulfide (DAS), a natural antioxidant found in garlic, induces Nrf2 activation and translocation in nuclei triggered by p38/ERK-signaling pathways in lung MRC-5 cells [44].* In vitro *experiments show that DS, KA, FGS, and CF exert their anti-inflammatory effects by activating Nrf2 and inducing Nrf2-regulated genes, including GCLC, NQO1, and HO1. Activation of Nrf2 occurs without reactive oxygen species production [41–43].

An oriental remedy called baicalein (BE) has shown to augment the Nrf2/heme oxygenase-1 (HO-1) pathway and to inhibit NF-*κ*B activation in LPS-instilled rat ALI models, thereby attenuating the histopathological symptoms of ALI [45]. Garlic* (Allium sativum)*, a member of the lily family, is a known antioxidant. DAS, enriched in garlic, is a natural organosulfur compound that prevents oxidative stress. DAS induces Nrf2 activation and translocation in nuclei triggered by ERK/p38 signaling pathways in lung MRC-5 cells [44]. Aged red garlic extract (ARGE), which has a more powerful antioxidant effect without the intense taste and smell of regular garlic, reduces the production of LPS-induced nitric oxide in macrophage. The polyphenolic and organosulfur compounds in ARGE could cause activation of Nrf2, eventually increasing HO-1 [46]. In a recent study we showed that glycosylation of aesculin (3-O-*β*-d-glycosyl aesculin) significantly suppressed neutrophilic lung inflammation in a mouse model of ALI. The anti-inflammatory function of glycosylated aesculin was mediated through Nrf2. In a mouse model of sepsis, a major cause of ALI, 3-O-*β*-d-glycosyl aesculin significantly enhanced the survival of mice, compared with aesculin, suggesting that glycosylation could confer the ability to activate Nrf2 on aesculin, enhancing the anti-inflammatory function of aesculin. Kaurenoic acid (ent-kaur-16-en-19-oic acid: KA) is a key constituent found in the roots of Aralia continentalis Kitagawa (Araliaceae) and has shown to be an Nrf2 activator. In a murine model of lung injury, we showed that KA has therapeutic potential against inflammatory lung disease, the effect of which is associated with Nrf2 activation.

Curcumin (or diferuloylmethane), a natural product found in turmeric, has been shown to inhibit multiple proinflammatory pathways and to activate anti-inflammatory pathways [51]. Curcumin modulates the activity of several transcription factors (e.g., NF-*κ*B, PPAR*γ* [peroxisome proliferator-activated receptor gamma], and activator protein 1). It inhibits TREM-1 in bone marrow macrophages. Curcumin also inhibits p300-HAT, a key epigenetic element known to activate transcription of the genes that regulate inflammation. Curcumin exhibits antioxidant effects at the level of the KEAP1–Nrf2 complex, resulting in the dissociation of KEAP1 and Nrf2, followed by nuclear accumulation of Nrf2. Kang et al. [50] showed that curcumin activates PI3K and p38 and increases AR activity, which may be a meaningful cellular response against oxidative stress.

Six different phase 1 human trials found no toxicity from curcumin. Both human and laboratory studies have found evidence of anti-inflammatory properties of curcumin, and it inhibits a bevy of enzymes and mediators of inflammation [38, 52]. The benefits of curcumin in sepsis patients appear to be mediated by the upregulation of PPAR-*γ*, leading to the suppression of the expression and release of TNF-*α* [39].

## 5. Conclusions

Since its first description 50 years ago, there has been an increase in the understanding of molecular pathogenesis and pathophysiology for the development of ARDS. However, to date the best practice involves “lung-protective ventilation” in mechanically ventilated patients with ARDS with no specific therapies directed towards lung inflammation. The inflammatory response in patients with ARDS is initiated, amplified, and modulated by a complex network of proinflammatory signaling pathways and oxidant stress generated by a variety of cell types in the lungs. Here, we reviewed some natural products whose biological effects may be useful in the development of new therapies for ARDS. Many of these agents have pleiotropic effects, such as inhibiting proinflammatory signaling while activating antioxidant defense mechanisms. One of the benefits of these natural products is that they have been consumed in the Asian subcontinent for centuries with no significant toxicity. However, to be developed for therapies, systematic studies (including pharmacokinetics and pharmacodynamics) must be carried out in human trials.

Although great strides have been made in the last several decades in defining molecular pathways for ALI and ARDS, these discoveries have not been translated into actual changes in medical treatments for patients with ARDS. To date, supportive strategies and lung-protective ventilation are the only approaches that have been shown to improve outcomes in these patients. A major challenge in generating effective therapeutics has been the ability to develop reliable animal models of critical illness that allow for the generation and testing of novel hypotheses and, ultimately, the translation of these findings to the human condition [53]. Areas of potential study include (1) novel methods of administration for better absorption (e.g., nanomicelles, lipid spheres) [54–56], (2) a combinatorial approach: multiple remedies could be administered simultaneously, as they are relatively low-toxicity products, and (3) use of natural products for prevention in high-risk patients (identified by lung injury prediction score).

## 6. Expert Commentary

The prognosis of patients with ARDS continues to be abysmal, with mortality rates ranging from 30% to 40%. Therefore, ARDS represents an unmet medical need and there is an urgent need to develop new therapies to treat patients with this condition. To date, treatment of the inciting event, lung-protective ventilation with lower tidal volumes, and optimal management of fluids remain the key therapeutic strategies for ARDS, but no specific therapies yet exist. Because of the complex nature of the disease (i.e., its involvement of multiple signaling pathways), neither blocking individual proinflammatory cytokines with antibodies nor the use of antioxidants has been rewarding. An interest in natural therapies as anti-inflammatory and antioxidative agents for systemic conditions has been growing. Given the complexity of the pathogenesis of ARDS, many natural products have been tested as pleiotropic agents that may help combat the inflammation and promote healing of the lung. We have reviewed the* in vitro *and* in vivo *data for many products that have been studied in preclinical models of ARDS.

The challenge lies in conducting translational studies to prove the efficacy and safety of these compounds in clinical trials. Although many of the aforementioned agents are widely consumed as herbal supplements or food additives in Asian countries, further study is needed before they can be adopted as therapies for ARDS. The pharmacokinetics and pharmacodynamics of these compounds in the setting of ARDS need to be established. The systemic administration of these compounds can also be challenging, so novel approaches to administer these compounds as nanomedicine or through aerosolization are other potential avenues for future study. Translational studies using these agents in patients with ARDS will provide potential opportunity to develop much-needed novel therapies for this devastating disease.

## Figures and Tables

**Table 1:** Natural products that decrease inflammation through NF-*κ*B pathway.

Natural product	Isolation	Study performed
Curcumin	Root of plant *Curcuma longa*	BMDM, mice [19, 20]
Ginsenoside Rg5	Rhizome of *Panax ginseng *C. A. Meyer	Macrophage, mice [21]
Alpinetin	Roots of *Alpinia Katsumadai Hayata*	RAW 264.7^*∗*^, mice [22]
PCA	Major benzoic acid derivative found in vegetables, nuts, brown rice, fruits, and herbal medicines	Mice [23]
LicoA	Flavonoid found in licorice root *(Glycyrrhiza glabra)*	RAW 264.7^*∗*^, mice [24]
Rosmarinic acid	Herbal plants including rosemary *(Rosmarinus officinalis)*, oregano *(Origanum vulgare)*, and spearmint *(Mentha spicata)*	Mice [25]
HYSA	Chinese herbal medication, *Carthamus Tinctorius L*. (safflower)	Mice [26, 27]
Linalool	Component of essential oils in several aromatic plants	RAW 264.7^*∗*^, mice [28]
PA	Pogostemon cablin	Mice [29]
Shikonin	Napthoquinone pigment extracted from root of *Lithospermum erythrorhizon*	Mice [30]

BMDM, bone marrow-derived macrophage; HYSA, hydroxysafflor yellow A; LicoA, licochalcone A; PA, patchouli alcohol; PCA, protocatechuic acid; ^*∗*^RAW 264.7, mice macrophage cell line.

**Table 2:** Natural products that decrease inflammation in in vivo model of ALI/ARDS.

Natural product	Isolation	Study performed
Honokiol	Component of the genus *Magnolia*	Mice [31]
ISOF	*Coleus forskohlii *native of Yunnan	Mice, rats [32]
Sophorolipid	Fermentation of *Candida bombicola*	Rats [33]
CAPE	Extract of propolis	Rats [34]
Ruscogenin	*Ruscus aculeatus*	Mice [35]
Bark extract of *Bathysa cuspidata*	*Bathysa cuspidata *(A. St.-Hil.) Hook f.	Rats [36]
CJT	Herbal remedy	Mice [37]

CAPE, caffeic acid phenethyl ester; CJT, *Callicarpa japonica Thunb*; ISOF, isoforskolin.

**Table 3:** Natural products that decrease oxidative stress through Nrf2 activation.

Natural product	Isolation	Study performed
Curcumin	Root of plant *Curcuma longa*	BMDM, mice [38, 39]
DS	Herbal formula in Korea (combination of 9 species of herbal plants)	RAW 264.7^*∗*^, mice [40]
KA	Dried roots of *Aralia continentalis*	RAW 264.7^*∗*^, [41]
FGS	Herbal formula in Korea	RAW 264.7^*∗*^, mice [42]
CF	Purified aqueous extract used in Asian medicine to treat blood stagnation	Mice [43]
DAS	Garlic extract *(Allium sativum)*	MRC-5 lung cells [44]
BE	Root of *Scutellaria baicalensis Georgi*, a Chinese herb	Rats [45]
ARGE	*Allium sativum, *a member of the lily family	RAW 264.7^*∗*^, [46]

ARGE, aged red garlic; BE, baicalein; BMDM, bone marrow-derived macrophage; CF, carthami flos; DAS, diallyl sulfide; DS, Dangkwisoo-san; FGS, fruit hull of *Gleditsia sinensis*; KA, ent-kaur-16-19-oic acid; ^*∗*^RAW 264.7, mice macrophage cell line.

## Abbreviations

AA: Arachidonic acid
ALI: Acute lung injury
AP1: Activating protein 1
AR: Aldose reductase
ARDS: Acute respiratory distress syndrome
ARE: Antioxidant response element
ARGE: Aged red garlic extract
BE: Baicalein
CF: Carthami flos
cAMP: Cyclic adenosine monophosphate
CAPE: Caffeic acid phenethyl ester
CAT: Catalase
CJT: * Callicarpa japonica Thunb*
COX 2: Cyclooxygenase 2
DAP12: TYRO protein tyrosine kinase binding protein
DAS: Diallyl sulfide
DS: Dangkwisoo-san
ERK: Extracellular signal-regulated protein kinase
FGS: Fruit hull of* Gleditsia sinensis*
GCLC: Glutamate-cysteine ligase, catalytic subunit
GPx: Glutathione peroxidase
HLJDT: Huang-Lian-Jie-Du-Tang
HMGB1: High-mobility group box 1
HO-1: Heme oxygenase- 1
HSYA: Hydroxysafflor yellow A
H3K4: Histone 3 and histone 4
I*κ*B*α*: Nuclear factor of kappa light polypeptide gene enhancer in B-cells inhibitor, alpha
IKK: I*κ*B kinase
iNOS: Inducible nitric oxide synthase
IRAK-4: Interleukin-1 receptor-associated kinase 4
ISOF: Isoforskolin
JNK: c-Jun-terminal kinase
KA: Ent-kaur-16-19-oic acid, or kaurenoic acid
KEAP1: Kelch-like ECH-associated protein 1
LicoA: Licochalcone A
LPS: Lipopolysaccharide
MAPK: Mitogen-activated protein kinase
MCP-1: Monocyte chemoattractant protein-1
MYD-88: Myeloid differentiation primary response gene 88
NF-*κ*B: Nuclear transcription factor-kappa B
NQO1: NAD(P)H quinine oxidoreductase- 1
Nrf2: Nuclear factor erythroid 2-related factor 2
PA: Patchouli alcohol
PCA: Protocatechuic acid
PPAR*γ*: Peroxisome proliferator-activated receptor gamma
ROS: Reactive oxygen species
SOD: Superoxide dismutase
TF: Tissue factor
TIR: Toll/interleukin-1 receptor
TIRAP: Toll/interleukin-1 receptor domain containing adaptor protein
TLR4: Toll-like receptor 4
TNF: Tumor necrosis factor
TRAF6: Tumor necrosis factor receptor-associated factor 6
TREM1: Triggering receptor expressed on myeloid cells 1
VCAM1: Vascular cell adhesion molecule 1.
